# Poly-substance use and antisocial personality traits at admission predict cumulative retention in a buprenorphine programme with mandatory work and high compliance profile

**DOI:** 10.1186/1471-244X-11-81

**Published:** 2011-05-12

**Authors:** Leif Öhlin, Morten Hesse, Mats Fridell, Per Tätting

**Affiliations:** 1 Department of Psychiatry, St Lars Hospital, Lund, Swedena; 2 Center for Alcohol and Drug Research, University of Aarhus, Copenhagen, Denmark; 3 Professor, Department of Psychology, Lund University & Linnaeus University, Växjö, Sweden; 4 Department of Psychiatry, St Lars Hospital, Lund, Sweden

**Keywords:** Buprenorphine, mandatory work, compliance, predictors, antisocial personality disorder, poly-substance

## Abstract

**Background:**

Continuous abstinence and retention in treatment for alcohol and drug use disorders are central challenges for the treatment providers. The literature has failed to show consistent, strong predictors of retention. Predictors and treatment structure may differ across treatment modalities. In this study the structure was reinforced by the addition of supervised urine samples three times a week and mandatory daily work/structured education activities as a prerequisite of inclusion in the program.

**Methods:**

Of 128 patients consecutively admitted to buprenorphine maintenance treatment five patients dropped out within the first week. Of the remaining 123 demographic data and psychiatric assessment were used to predict involuntary discharge from treatment and corresponding cumulative abstinence probability. All subjects were administered the Structured Clinical Interview for DSM-IV-TR, and the Symptom Checklist 90 (SCL-90), the Alcohol Use Disorder Identification Test (AUDIT), the Swedish universities Scales of Personality (SSP) and the Sense of Coherence Scale (SOC), all self-report measures. Some measures were repeated every third month in addition to interviews.

**Results:**

Of 123 patients admitted, 86 (70%) remained in treatment after six months and 61 (50%) remained in treatment after 12 months. Of those discharged involuntarily, 34/62 individuals were readmitted after a suspension period of three months. Younger age at intake, poly-substance abuse at intake (number of drugs in urine), and number of conduct disorder criteria on the SCID Screen were independently associated with an increased risk of involuntary discharge. There were no significant differences between dropouts and completers on SCL-90, SSP, SOC or AUDIT.

**Conclusion:**

Of the patients admitted to the programme 50% stayed for the first 12 months with continuous abstinence and daily work. Poly-substance use before intake into treatment, high levels of conduct disorder on SCID screen and younger age at intake had a negative impact on retention and abstinence.

## Background

A large proportion of patients with substance dependence relapse during or after treatment [1-3]. Identifying predictors of the risk of relapse in different treatment models may provide valuable information about what type of patients need extra services to obtain a satisfactory result in treatment.

In treated samples psychosocial factors, such as peer-group relationships, family problems, employment, and social support, predict relapse to opiate use [4]. In an older meta-analysis of predictors of relapse to opiate use, it was found that a high level of pre-treatment drug use, a history of prior treatment, no prior abstinence from opiates, abstinence from alcohol, depression, high stress, employment problems, association with substance abusing peers, short length of treatment, and leaving treatment prior to completion were all associated with relapse [5]. Combined effect sizes were generally small. A frequently reported important predictor of relapse is the number of substances in baseline urine toxicology [e.g. [6,7]].

Another potentially important factor is the presence or absence of an antisocial personality disorder. Conduct disorder (DSM IV-TR) is a precursor of anti-social personality disorder and a childhood or adolescent CD develops into an adult ASPD in between 30% and 50% of all cases [8]. A recent meta-analysis found that antisocial personality disorder is a complex predictor of outcome. In settings such as therapeutic communities antisocial personality disorder was a positive factor in predicting retention, whereas in other types of treatment, such as outpatient drug-free counselling, it was a negative predictor [9]. Along similar lines, Daughters and colleagues found that antisocial patients who were under legal supervision had better retention in inpatient treatment compared with patients without an antisocial personality disorder who were also under legal supervision, and those who were not under legal supervision. Antisocial patients without legal supervision had the poorest retention rates [10]. Thus, the significance of antisocial personality disorder may be dependent on the type of structure provided. The influence of other personality disorders on retention and outcome is less well known.

In the review of the international literature on evidence-based treatment of substance abuse, Berglund et. al. concluded that a) a focus on the substance use, b) high treatment structure, c) continuous intervention lasting for at least three months and d) a focus on comorbidity was associated with effective treatment interventions in comparison with less effective interventions [11].

One potential predictor that has been studied little in patients with drug addiction is sense of coherence [12]. The theory of sense of coherence was introduced by American-Israeli medical sociologist Aaron Antonovsky, who developed the Sense of Coherence Scale (SOC). Sense of coherence is believed to be a global orientation to the world and the personal environment as comprehensible, manageable, and meaningful. Antonovsky claimed that sense of coherence has a significant positive influence on health. Research generally supports that the SOC is moderately stable over many years and has predictive validity for physical and mental health, after controlling for baseline health [12].

The few studies that have been conducted concerning the impact of the sense of coherence in substance-dependent populations have generally yielded relatively strong relationships between higher sense of coherence and improvement in substance use problems [13,14], or lower mortality rates during follow-up [15,16].

The aim of this study was to study predictors of cumulative retention in a consecutive cohort of buprenorphine-treated patients with the particular emphasis on elements reinforcing structure of treatment. Based on the literature, we assumed that an indication of antisocial personality disorder, here operationalised by the number of criteria endorsed for conduct disorder on the SCID Screen, poly-substance abuse at baseline as measured by the number of positive urine samples for different illicit drugs in urine analysis at intake, and severity of self-reported general psychiatric distress (Global Severity Index) at baseline on the Symptom Checklist 90 (SCL-90), and extent of subjective sense of coherence (total raw score) were predictive of attrition from treatment. It was also hypothesised that a low level of personality pathology on SSP and a low consumption of alcohol as measured by AUDIT (total raw score) would be associated with high retention.

## Methods

The study was based on data from a prospective study of the course of buprenorphine treatment in a highly structured clinic. Patients in the clinic received maintenance treatment for opiate dependence, either buprenorphine alone or buprenorphine/naloxone formulation tablets to be taken sublingually.

The subjects in the study were consecutively admitted for treatment between August 2004 and November 2009. At intake to treatment, patients were informed of the conditions of treatment and, after both verbal and written consent, were requested to provide a urine specimen, and were seen by a senior consultant psychiatrist who initiated and supervised the buprenorphine treatment continuously. The treatment staff comprising nurses and a social worker supervised the daily activities as well as the structure and contacts with other authorities responsible for the treatment. There is a continuous and close contact between the patients and the staff. Work activities and education were organized through joint collaboration between representatives from the social insurance, social welfare, employment agency and the psychiatric unit at the hospital. This type of collaboration in a maintenance programme is unique in Sweden.

All subjects who completed at least 4 weeks of treatment and who agreed to be included in the firm structure of the programme were enrolled. According to the regulations from the National Board of Health and Welfare (2007) [17] the exclusion criteria for opioid substitution treatment, and thus for the study were as follows: being younger than twenty years of age, less than one year of frequent opiate use, florid symptoms of psychosis/history of psychosis or ongoing compulsive treatment within psychiatry [18].

After completing detoxification the subjects went through a phase of psychological testing and psychiatric assessment including psychiatric screening for psychiatric symptoms and personality disorders: (SCID-II), SCL-90, AUDIT, SSP, SOC and a standardized clinical interview. ICD-10 diagnoses of substance disorder were issued for all patients admitted. In addition diagnoses of psychiatric disorders were issued in relation to additional pharmacological treatment interventions.

The subjects were followed from their admission to treatment and until they were involuntarily discharged, or until January 1, 2010. In addition to the supervised urine samples interviews and tests were repeated every third months up to one year after admission.

### Treatment context

The Buprenorphine clinic is part of the St. Lars psychiatric hospital in Southern Sweden, Scania County with an uptake area of the entire Southern region of Sweden. Treatment is free for the patients. Patients first attend a meeting with the unit psychiatrist (PT), the clinic social worker (LÖ), and a clinic attendant or nurse. Patients are then offered treatment at the clinic on the basis of mutual agreements during this meeting and are encouraged to begin tapering their use of substances before admittance for treatment.

The clinic employs abstinence-oriented Buprenorphine maintenance treatment, in the sense that no illegal drug use is tolerated after admission to the program. Patients in Buprenorphine treatment are discharged from treatment if the rules are violated. Violence of all kinds in the unit, directed at staff or fellow patients, is prohibited, as well as purchasing or dealing drugs during treatment. Criminal activities result in discharge from the program. The patients must adhere to the ongoing social and medical case management within the clinic. This includes participating in drug counselling at their home town's counselling services, mostly case-management or cognitive behavioural therapy or a Twelve Steps approach. The amount of counselling is decided by the home town services.

Being discharged from the program requires that the positive urine screen at the unit is verified by an independent laboratory finding. Urine samples are collected under surveillance and sent to Lund University Hospital's chemical laboratory. If tests are positive for drugs, they are sent to a second laboratory for a confirmatory analysis. Urine samples are analyzed using Gas chromatography-mass spectrometry (GC-MS) [19]

Discontinuation of treatment is always decided jointly by the senior consultant psychiatrist and the staff after informing the other authorities and the patient. After three months of suspension the patient may apply for a renewed treatment. During the suspension period the patient is seen on an outpatient basis. The aim of that particular strategy is to maintain contact with the patient in order to reduce the risk of drug overdose. The patient is also allowed to continue in his work/education.

The staff, outpatient counsellors and officials from social services and from the regional social insurance office together help the patients to find work, and to coordinate their work with treatment adherence. All patients submit three tests per week, and maintain a fulltime job or fulltime study. After 4 months of treatment, the required urine tests are reduced to one per week.

All patients who are admitted are administered self-report tests at intake (see measures below). When patients score two standard deviations above the age and gender adjusted norms on the Alcohol Use Disorder Identification Test (AUDIT), they are routinely offered pharmacotherapy for alcoholism, generally disulfiram or acamprosate. Patients scoring above T = 70 on Symptom Checklist 90 (SCL-90) at any time are referred for a full psychiatric assessment and may be offered pharmacotherapy indicated.

During the ongoing treatment patients with non-treatable adverse reactions to buprenorphine are referred to the general opioid agonist maintenance unit at the same hospital, where methadone is an alternative intervention.

After one month of treatment, patients undergo assessment for personality disorders with the SCID-II and SSP (see below). Thus, all patients who are administered the SCID-II have been drug free for one month.

### Assessments

At intake to treatment patients in the study were asked to complete the Alcohol Use Disorder Identification Test (AUDIT), the Sense of Coherence scale (SOC), and the Symptom Checklist 90 (SCL-90). After one month of treatment, patients were administered the Structured Clinical Interview for the DSM-IV-TR (SCID-II) and the Swedish universities Scales of Personality (SSP). The SOC and SCL-90 tests were repeated every third month and AUDIT twice during the first year of study.

#### The SCID-II and SCID Screen

The Structured Clinical Interview for the DSM-IV- TR, Axis II (SCID-II) is a widely used semi-structured interview designed to assess personality disorders [20]. The interview covers the eleven DSM-IV Personality Disorders (including personality disorders not otherwise specified) and the appendix categories Depressive Personality Disorder and Passive-Aggressive Personality Disorder. Patients first complete the self-report questionnaire and in a subsequent interview the interviewer asks follow-up questions about items that are endorsed on the questionnaire. For antisocial personality disorder the SCID-II screen contains questions about conduct disorder before age 15. If patients satisfy criteria for conduct disorder, they are asked questions about all criteria for adult antisocial personality disorder.

For the present study the symptom count from the SCID screen for conduct disorder was used as indicators of personality disorder-related traits. While there are advantages with the full interview data for clinical use (the ability to have a dialogue with the patient and understand the subjective meaning of the problems reported), the SCID-questionnaire is less susceptible to interviewer bias and has been shown to be highly correlated with symptom counts from the interview with a correlation of 0.86 between the questionnaire and interview [21], and to be highly stable in drug abusers, with a test-retest correlation of 0.76 over one year [22].

#### The Symptom Checklist 90 - SCL-90

The Symptom Checklist-90 (SCL-90) is a self-report measure of psychiatric symptoms, covering nine different symptoms relating to psychiatric conditions. Symptoms are rated on a 5 point Likert scale [23]. The patient responds to each statement (*e.g*., "nervousness or shakiness") to what degree of severity the symptom has been present in the past week on a 5-point scale (0 "not at all", 1 "a little bit", 2 "moderately", 3 "quite a bit", or 4 "extremely"). For the calculations only the Global Severity Index, the mean of all items, was used.

The Swedish SCL-90 version was translated and back-translated into English, and standardized on a nationally representative sample of 5,000 community residents and validated against psychiatric samples with relevant diagnoses and substance abusers (total n = 1,800). On the basis of the representative sample gender-adjusted T-scores have been developed. T-scores have a normal mean of 50 and a standard deviation of 10 [24]. The cut-off level indicating clinically significant problems was set to T≥70. These are reported in the descriptive statistics for the sample.

#### The Sense of Coherence Scale (SOC)

The Sense of Coherence Scale is a 29-item self-report scale designed to measure Antonovsky's construct of sense of coherence [11]. It is designed to measure a basic attitude to life, or a personality dimension, hypothesized to facilitate the ability to cope with stress. The Swedish standardization and validation is based on Hansson and Olsson [25].

#### The Alcohol Use Disorder Identification Test (AUDIT)

The AUDIT is a 10-item scale designed to measure alcohol related disorders [26] used in a very large number of both epidemiological and clinical studies. For this study we report age- and gender-adjusted T-scores based on a Swedish standardization study [27]. However, for statistical analyses, we used the unadjusted scores, since the subjects' age and gender were also included as co-variates.

#### The Swedish universities Scales of Personality (SSP)

The Swedish universities Scales of Personality (SSP) is a revision of the Karolinska Scales of Personality (KSP). SSP is published in Sweden but has been translated into English [28]. The personality profile is presented in T-score format (mean 50 and standard deviation 10). It has 91 items and yields 13 personality scales: somatic trait anxiety, psychic trait anxiety, stress susceptibility, lack of assertiveness, impulsiveness, adventure seeking, detachment, social desirability, embitterment, trait irritability, mistrust, verbal trait aggression and physical aggression.

### Statistical analysis

All statistics were calculated on Stata 11 for Windows. Cox Proportional Hazard Regression was used to assess predictors of cumulative retention. All selected predictors (age, gender, number of drugs in urine at baseline, AUDIT score, criteria count for conduct disorder from the SCID Screen and SCL-90 global severity index) were entered in a multivariate analysis. Two patients who dropped out within the first two days of treatment were treated as censored observations. We controlled for age and gender, because two of our covariates are known to vary substantially by age and gender, namely psychiatric symptoms [29] and antisocial behaviour [30,31]. We first estimated a model for each covariate to describe the univariate relationship between the covariate and retention. Further, the proportional hazards assumption for each covariate was tested. The test is a χ^2 ^statistic with one degree of freedom, where rejection of the null hypothesis indicates that the effect of a covariate is not constant over time.

Because there is evidence that dimensional models of antisocial personality pathology are superior to taxonomic ones, we chose to enter the criteria count rather than a categorical predictor based on a rationally derived cut-off for diagnosis that would result in loss of information on either side of the cut-off [32-34]. For the statistical predictor analysis raw scores were used.

Ethics approval was obtained from the Regional Ethical Review Board in Lund (# 847/2004).

## Results

### Subjects

A total of 128 subjects were originally included. Five subjects either dropped out within the first weeks or did not stay long enough to complete the SCID-II and were excluded from further analyses, leaving 123 subjects. No statistical comparison of early dropouts with the remaining patients was deemed necessary.

Descriptive statistics are summarized in Table 1. Of the remaining subjects 97 were men and 26 were women. The mean age at admission was 33.5 (range: 22 to 62, SD = 8.6). The mean gender-adjusted T-score for the SCL-90 Global Severity Index (GSI = 81.3) was 3 standard deviations above the normative gender- and age-matched mean for the Swedish population. The mean SOC score was 119 (range: 64 to 191), one standard deviation below the norm group, and the mean number of personality disorders according to the SCID-II interview was 3.2 (range: 0 to 9). A total of 17 had no personality disorder, 39 had just one personality disorder, and the remaining patients had two or more. The most common personality disorders were antisocial (74%), narcissistic (56%), schizotypal (40%) and borderline personality disorder (37%).

**Table 1:** Descriptive statistics for the cohort at admission (n = 123)

	Mean or N	Standard deviation or %
Women	26	21%

Men	97	79%

Age at admission	33.2	8.5

High school completed	35	30%

***Symptom Checklist: SCL-90***		

Global Severity Index (GSI) T-score	81.8	24.1

SCL-90: Anxiety - T-score	79.8	23.3

SCL-90: Depression - T-score	76.1	21.8

AUDIT T-score	59.1	19.0

Antisocial personality disorder (SCID II)	93	74%

No personality disorder (SCID II)	17	13%

***Drugs detected in urine samples at admission***		

Amphetamine	17	14%

Benzodiazepines	60	49%

Buprenorphine	56	46%

Cannabis	43	35%

Cocaine	1	1%

Dextropropoxyphene	5	4%

Methadone	8	7%

Opiates	62	50%

Of all patients 67% scored below 60 on the AUDIT T-score, which indicates scores within the normal-range and 13% scored above 70 (i.e., two standard deviations above the age and gender-adjusted mean), indicating serious alcohol problems.

During the treatment 41 patients (33% of the whole group) developed psychiatric symptoms indicating need for additional pharmacological treatment with antipsychotic or/and antidepressant medication. The patients were prescribed olanzapine (11), mirtazapine (27), citalopram (2) and venlafaxine (1). The average T-scores for depression in the group undergoing pharmacological treatment was significantly higher: T = 80 (S.D. = 24.9) than in the group with no prescribed pharmacological treatment, T = 69 (S.D. = 19.8), validating the clinical diagnoses (t_121 _= 2.66, p < .001). In the group treated with these specific pharmacological interventions 25 patients of the 41 (61%) completed treatment over the first 12 months.

## Discharge and dropout from treatment

The observation period ranged from two weeks to 64 months. The median survival time was 13 months. In all, 61 patients (50%) remained in treatment for at least one year, 6 (5%) ended treatment on their own request and 56 (45%) were discharged involuntarily. Of the 56 patients who were involuntarily discharged 34 (30%) were readmitted for a new buprenorphine treatment after the suspension period and another 13 (11%) have started in the methadone maintenance program. One patient died after committing suicide 6 months after leaving treatment.

The results of the unadjusted and adjusted models are shown in Table 2. In the columns 2-4 hazard ratios with confidence intervals are shown from the unadjusted models. In column 5 the χ^2 ^for violation of the proportional hazards assumption is shown. None of the tests indicated that the assumption was violated. The tests SSP and SOC were dropped since there were no significant differences between completers and non-completers on those measures, and the amplitude of the T-scores were in general within the standard deviation on the subscale averages.

**Table 2:** Results of Cox Proportional hazard regression

	Hazard ratio^1^	Risk ratio 95% lower limit	Risk ratio 95% upper limit	Test of proportional odd assumption χ^2^(1)	Hazard ratio^2^	Risk ratio 95% lower limit	Risk ratio 95% upper limit	Z	P
SCL-90 **GSI**	1.27	0.94	1.73	0.83	1.25	0.88	1.78	1.26	0.21

AUDIT	1.00	0.96	1.04	1.01	0.96	0.92	1.00	-1.83	0.07

Female gender	1.65	0.81	3.36	0.06	1.57	0.71	3.44	1.12	0.27

Age	1.02	1.00	1.05	0.79	1.05	1.01	1.09	2.71	<0.01

CD count	1.10	1.01	1.20	0.00	1.12	1.02	1.23	2.30	0.02

No of drugs in urine	1.37	1.11	1.67	0.04	1.34	1.08	1.67	2.65	<0.01

The multivariate regression was significant (likelihood ratio X^2^_(5) _= 22.56, p < 0.002) for the variables: age, number of drugs in urine and on the conduct disorder screen. In the multivariate analysis, higher age, poly-substance abuse, and the number of conduct disorder criteria at intake were significantly associated with discharge before the ending of the first year.

**Figure 1:**
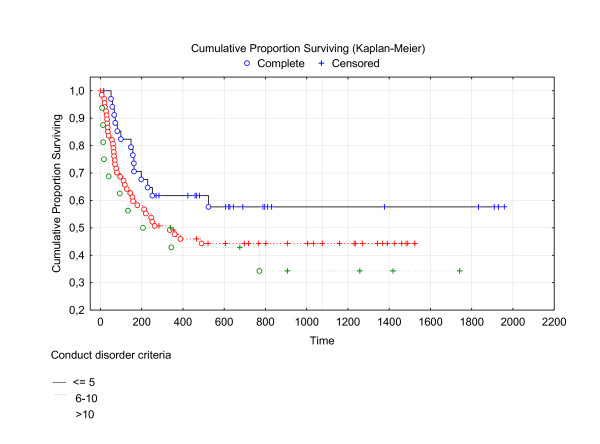
**Survival curve over the first 2000 days in patients with 0-4 criteria, 5-9 criteria and 10 or more criteria on Conduct disorder**.

The relationship is illustrated in Figure 1.

## Discussion

The program had a high retention rate compared to levels reported in other studies [35,36]. Fifty percent of the patients remained in treatment over the first year showing high compliance with the treatment goals demonstrated by negative urine specimens three times a week and continuous work attendance. In line with some previous research, baseline poly-substance use predicted poor response to opiate substitution treatment [6,7]. The number of drugs in urine at the time of treatment entry was significantly associated with drop-out from treatment. Poly-substance abuse at intake indicated problems staying abstinent over a prolonged period and increased the risk of discharge in this cohort.

It seems that strategies are needed to support patients who have a high degree of poly-substance abuse prior to entering treatment. Other types of treatments like methadone, residential treatment or alternative interventions may be indicated in some cases. However, it seems that the one-year level of abstinence associated with high compliance and good treatment response stands well in comparison to previous studies of drop-out and retention in substitution treatment [37].

In line with several other studies, the SCID screen as an indicator of antisocial traits had a significant impact on discharge from treatment in this study, even after controlling for a number of relevant covariates [10,38]. As noted in the introduction, a significant interaction may exist between structure and type of treatment and the impact of personality disorders in general and antisocial personality disorder in particular. The treatment in the clinic had a clear focus on abstinence, high structure, high compliance with the treatment regimen, and the contingency between work attendance and the continuance of treatment, a format that should be well suited for patients with co-morbid substance use disorder and antisocial personality disorder [9,10,39,40]. Even so, the patients with more severe antisocial personality traits, as measured by the number of conduct criteria endorsed, were at increased risk of dropping out of treatment.

Self-reported symptoms as measured by the SCL-90 were associated with higher but non-significant risk of involuntary discharge. The results from previous research have been mixed concerning the impact of depression and anxiety on involuntary discharge [41]. Patients staying in treatment for at least one year showed a statistical tendency of p < .10 on the SOC scale, but SOC was not predictive of treatment completion.

In a clinical context the findings suggest that a highly structured and stringently monitored opioid substitution treatment may be effective for a relatively wide group of patients with opiate dependence and a high level of psychiatric co-morbidity, including a very high prevalence of antisocial personality disorder [11]. The work module in this programme is of particular interest in this regard, since it is a unique way of increasing structure and providing a meaningful life situation for the patients. The level of retention in this study is equivalent to well functioning residential treatment programmes as described by Bell (1985) [40], and also comparable to levels of retention in high quality substitution programmes in the USA and in Europe [35,36].

### Strengths and limitations

The present study is based on a cohort of patients consecutively admitted for treatment. All patients who were admitted gave both written and verbal consent, and the data sets were almost complete. The use of well-validated instruments to assess conduct disorder and symptoms as well as the use of stringent criteria for treatment success increase the internal validity of this study.

As regards limitations, it is important to note that the patients in this study were self-selected for a treatment that is both abstinence-oriented and oriented towards full rehabilitation in an outpatient setting. Therefore, the results may not generalize to treatment modalities with other treatment goals and a less severe focus on abstinence. The size of the sample is another limitation, especially in terms of studying interactions between variables.

## Conclusions

The buprenorphine program in this study demonstrated a high level of retention over one year and beyond with a strict focus on abstinence and work adaptation. Younger patients and those who reported many symptoms of conduct disorder on the SCID-II screen as a proxy of anti-social personality disorder, had a higher dropout rate than other patients throughout the study.

## Competing interests

Conflict of interest declaration: The authors declare that they have no financial or other conflicts of interests in relation to this manuscript. The funders had no say with regard to the analyses, interpretation, or decision to submit the manuscript for publication.

## Authors' contributions

LÖ, MF and PT designed the study. LÖ organized the data collection and collected the data. MH carried out the statistical analyses and drafted the manuscript. LÖ, MH and MF wrote the final manuscript. All authors read and approved the final manuscript.

## Pre-publication history

The pre-publication history for this paper can be accessed here:

http://www.biomedcentral.com/1471-244X/11/81/prepub
